# Supplementary material for: Stabilizing atomic Ru species in conjugated sp2 carbon-linked covalent organic framework for acidic water oxidation
Source: Nat Commun. 2024 Jun 26;15:5419. doi: 10.1038/s41467-024-49834-5 (PMC11208516; doi:10.1038/s41467-024-49834-5)
Supplement: Supplementary file 1 — supporting information [file 41467_2024_49834_MOESM1_ESM.pdf]

Supplementary Information for

Stabilizing atomic Ru species in conjugated sp^2 carbon-linked covalent organic framework for acidic water oxidation

Hongnan Jia,[1,3] Na Yao,[2,3] Yiming Jin,[1] Liqing Wu,[1] Juan Zhu[1] and Wei Luo[1,*]

[1]College of Chemistry and Molecular Sciences, Wuhan University, Wuhan, Hubei 430072, P. R. China.

[2]State Key Laboratory of New Textile Materials and Advanced Processing Technologies, Wuhan Textile University, Wuhan, Hubei 430073, P. R. China.

[3]These authors contributed equally to this work.

*e-mail: wluo@whu.edu.cn

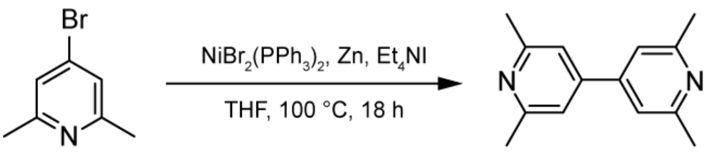

Supplementary Fig. 1 Synthesis of 2,2’,6,6’-tetramethyl-4,4’-bipyridine (TMBP).

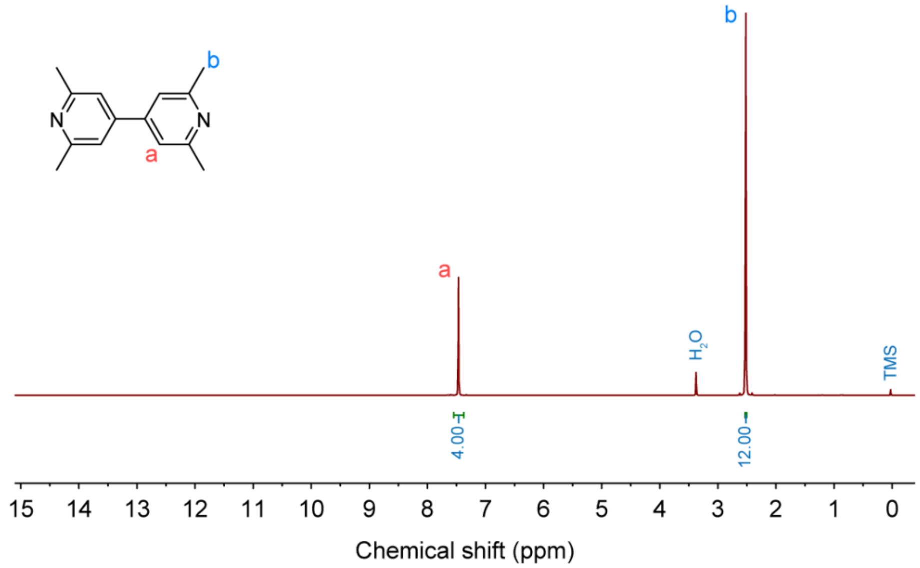

Supplementary Fig. 2 ^{1}H NMR spectrum of TMBP monomer recorded in DMSO.

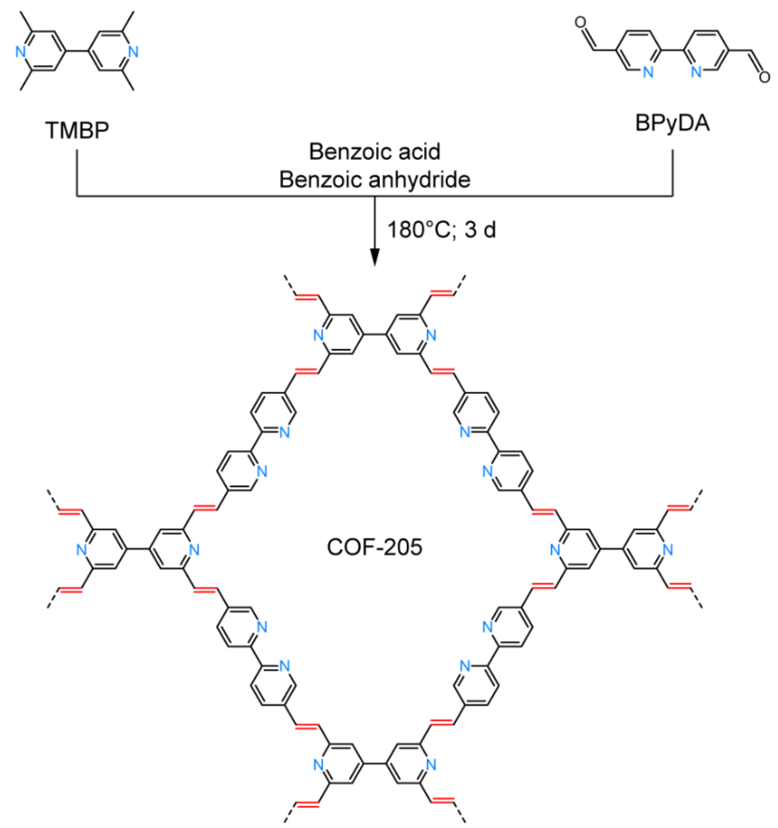

Supplementary Fig. 3 The structure illustration of the designed COF-205.

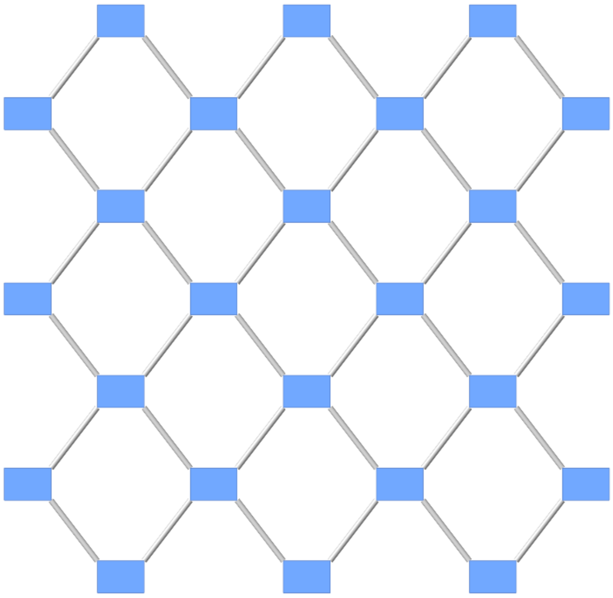

Supplementary Fig. 4 The ***sql*** topology of the COF-205 and COF-205-Ru.

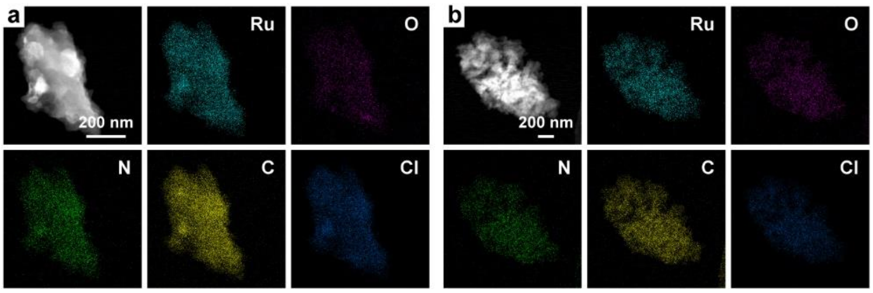

Supplementary Fig. 5. Element distribution analysis. TEM-EDS images of (a) COF-205-RuO_xCl_y and (b) COF-205-Ru.

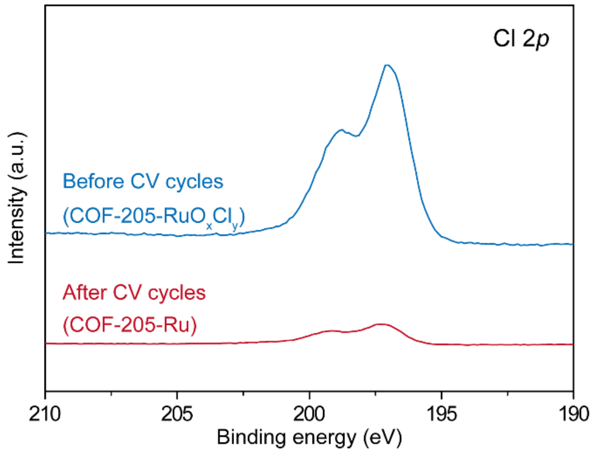

Supplementary Fig. 6. Cl 2*p* XPS spectra of COF-205-RuO_xCl_y and COF-205-Ru.

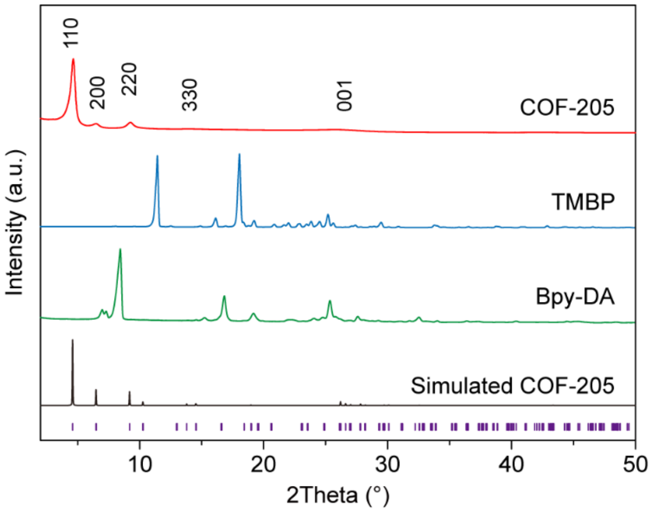

Supplementary Fig. 7 PXRD pattern of BPy-DA, TMBP and as-synthesized COF-205. Where green lines show Bpy-DA diffraction data; blue lines show TMBP diffraction data; red lines represent COF-205 diffraction data; black lines show the calculated patterns and purple bars show the Bragg position.

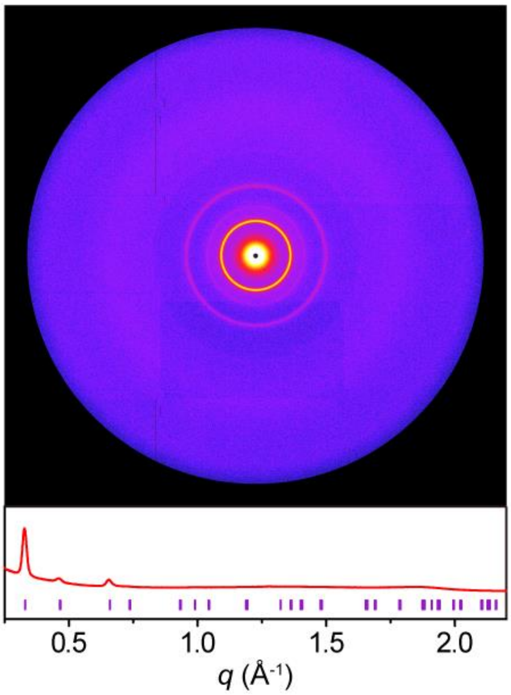

Supplementary Fig. 8 2D SAXS image and corresponding pattern of experimental SAXS data for the as-synthesized COF-205-Ru. Red line: experimental data; Purple line: Simulated Bragg position.

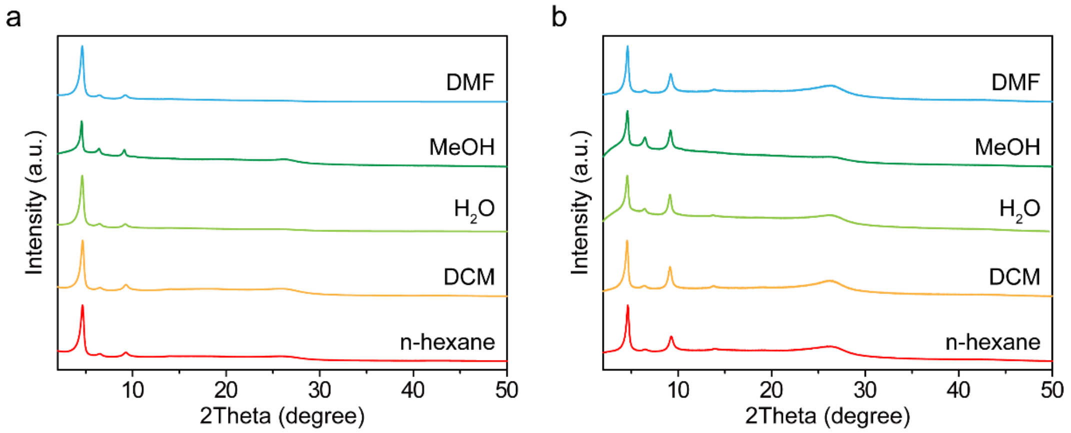

Supplementary Fig. 9 Solvent stability evaluation. PXRD patterns of (a) COF-205 and (b) COF-205-Ru samples after soaking in n-hexane, dichloromethane (DCM), H_2O, methanol (MeOH), or N,N'-dimethylformamide (DMF) for two weeks.

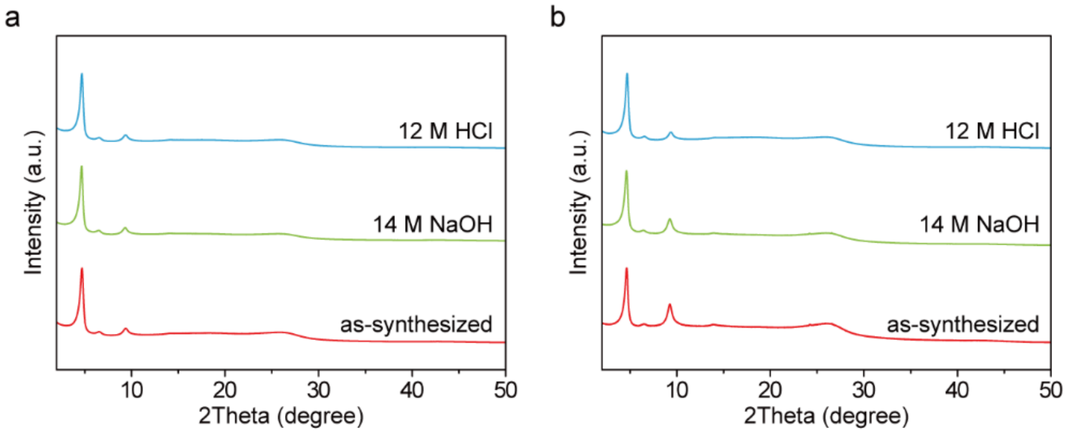

Supplementary Fig. 10 Acid-base stability evaluation. Comparison of the experimental PXRD patterns of (a) COF-205 and (b) COF-205-Ru samples with the diffraction patterns after immersion in HCl (12 M) or NaOH (14 M) aqueous solution for two weeks.

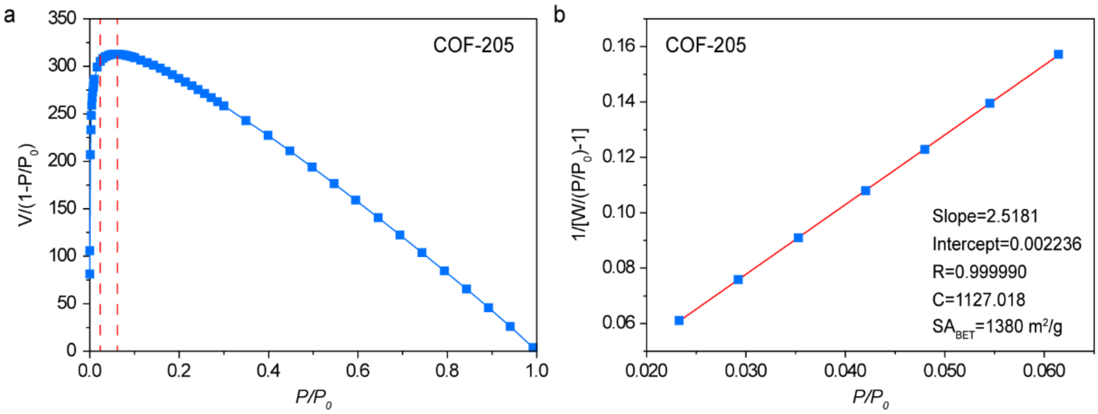

Supplementary Fig. 11 The BET surface area calculated for COF-205 based on nitrogen adsorption isotherm at 77 K. (a), Only points between the dashed lines are selected based on the first consistency criterion; (b), Plot shows good fitting to the BET model.

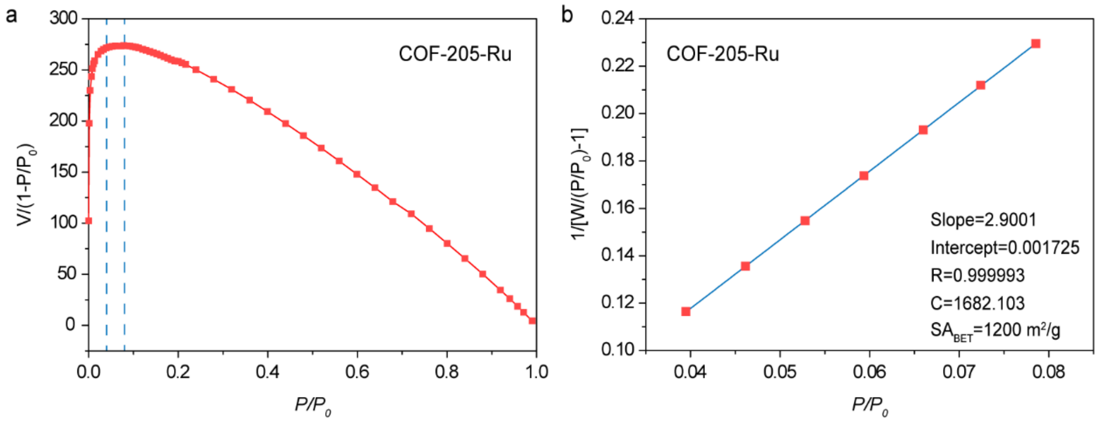

Supplementary Fig. 12 The BET surface area calculated for COF-205-Ru based on nitrogen adsorption isotherm at 77 K. (a), Only points between the dashed lines are selected based on the first consistency criterion; (b), Plot shows good fitting to the BET model.

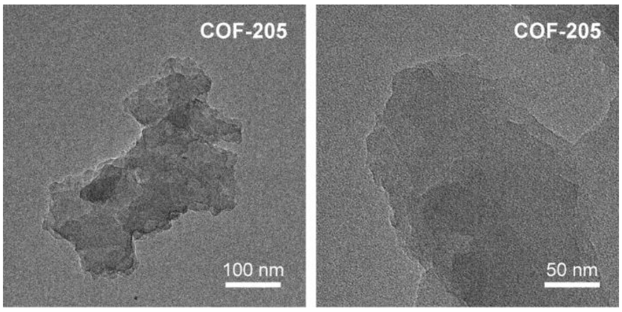

Supplementary Fig. 13 TEM images of COF-205.

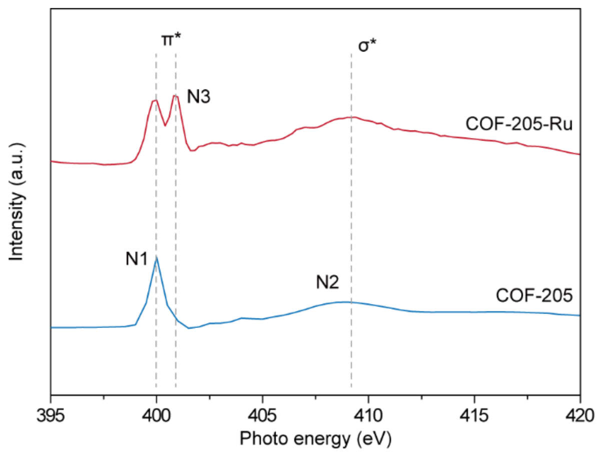

Supplementary Fig. 14 N K-edge XANES spectra of COF-205 and COF-205-Ru.

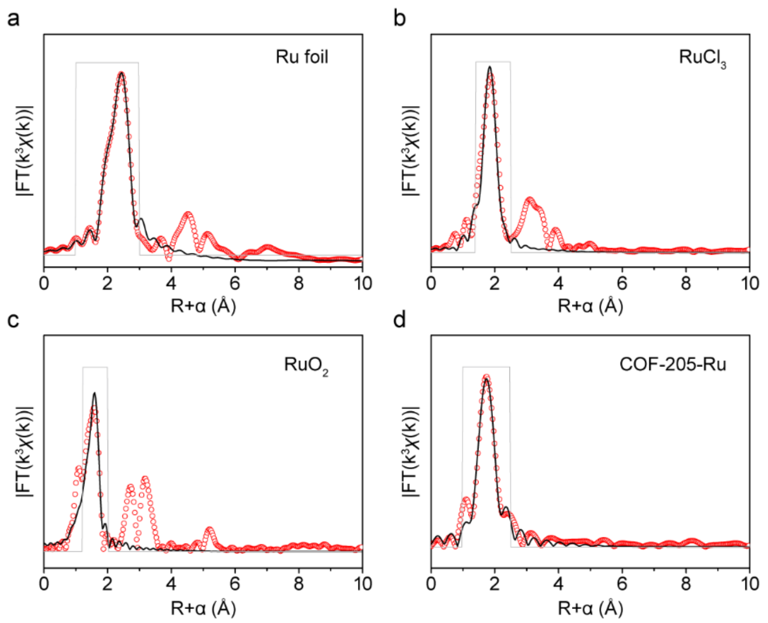

Supplementary Fig. 15 Ru K-edge EXAFS (point) and curvefit (line) shown in R-space. (a), Ru foil; (b), $RuCl_3$; (c), RuO_2 and (d), COF-205-Ru.

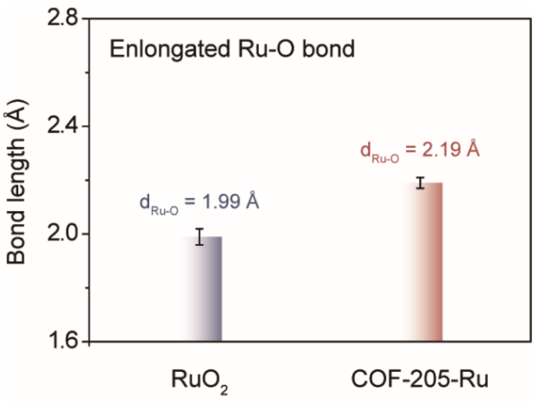

Supplementary Fig. 16 Comparison of Ru-O bond length for RuO_2 and COF-205-Ru.

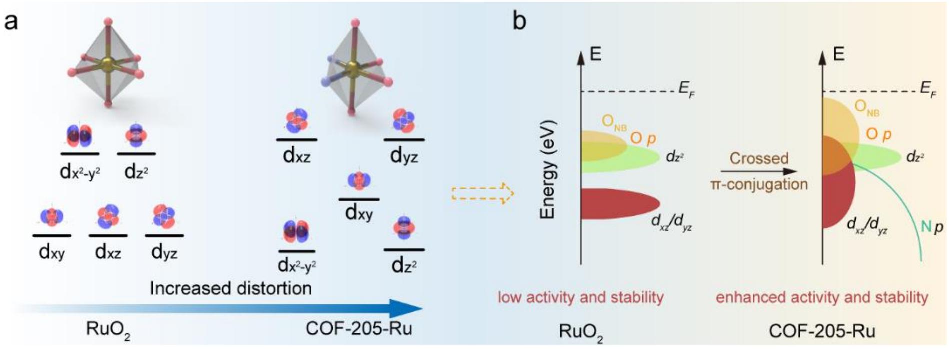

Supplementary Fig. 17 Illustration of influence of structural distortion on band structure. (a), Effect of ligand field variation on d-orbital energy level splitting; (b), Illustration of the larger hybridization degree between Ru-*d* orbital and O-*p* orbital.

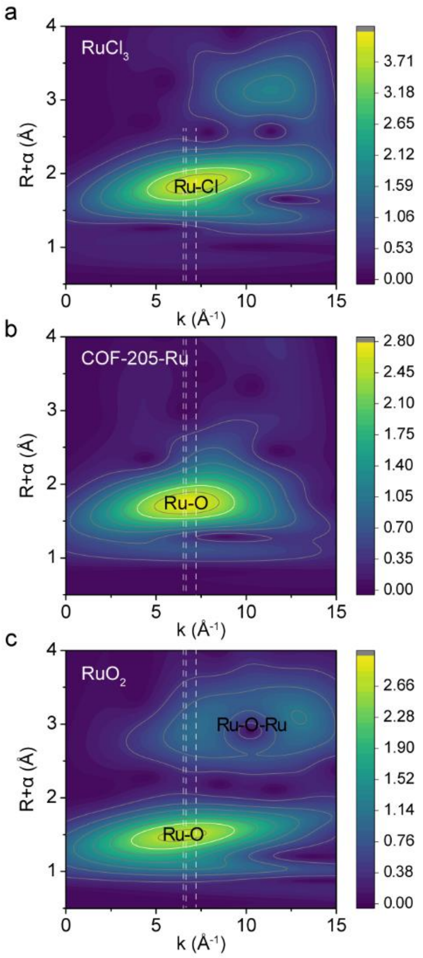

Supplementary Fig. 18 Wavelet transformation of samples. (a), $RuCl_3$; (b), COF-205-Ru and (c) RuO_2.

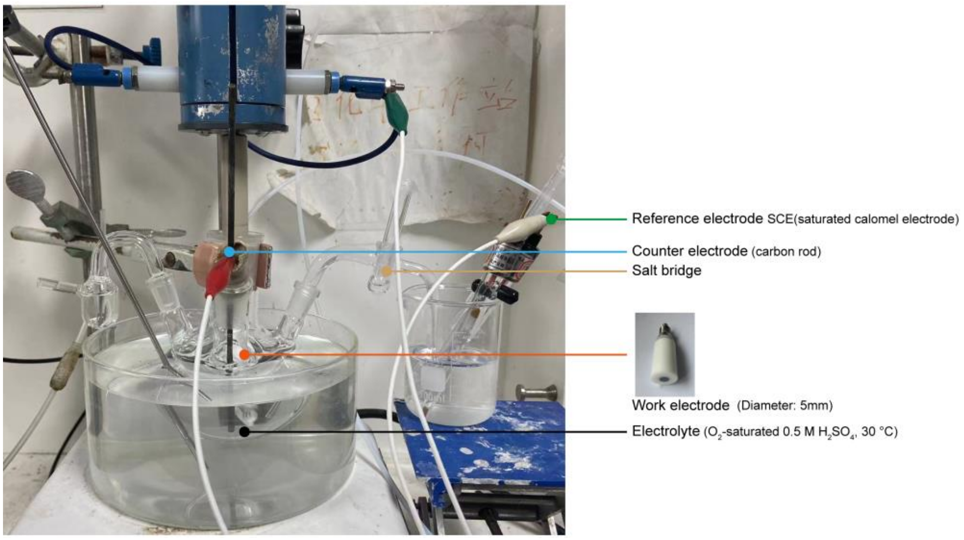

Supplementary Fig. 19 The electrochemical cells with three-electrode system for acidic OER.

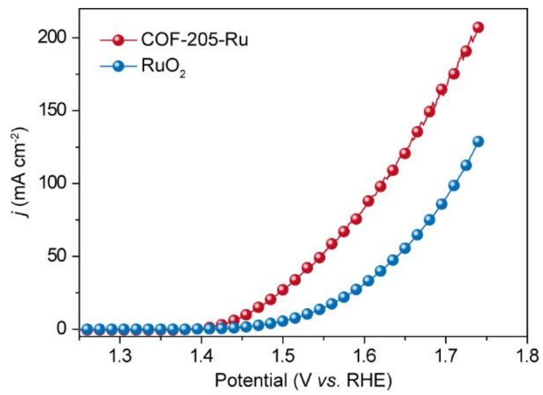

Supplementary Fig. 20 OER performance of COF-205-Ru and commercial RuO_2 on carbon cloth electrode under 0.5 M H_2SO_4 electrolyte (without *iR*-compensation).

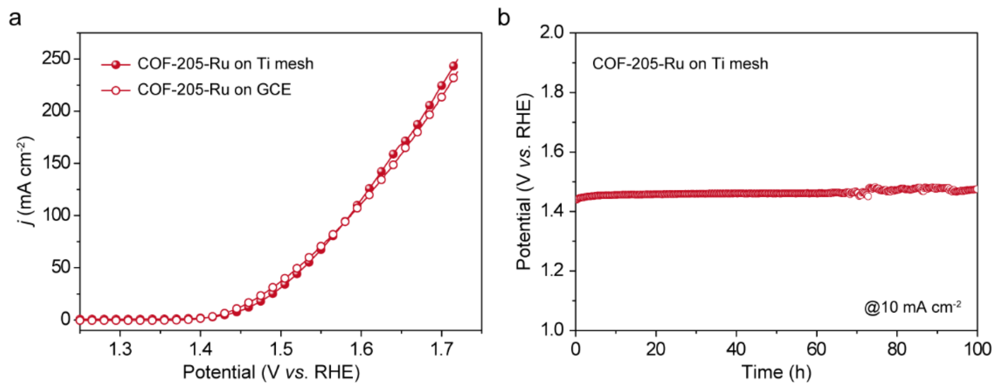

Supplementary Fig. 21 OER performance of COF-205-Ru on Ti mesh electrode under 0.5 M H_2SO_4 electrolyte (without iR-compensation). (a) Polarization curves; (b) Chronopotentiometry measurement at 10 mA cm^{-2}.

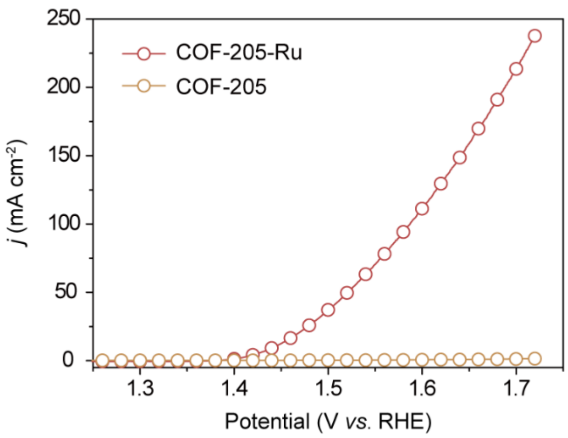

Supplementary Fig. 22 Comparison of OER catalytic performance for pure COF-205 and COF-205-Ru (without *iR*-compensation).

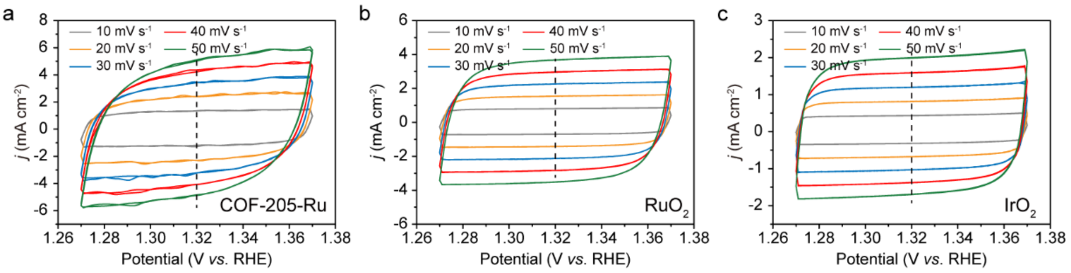

Supplementary Fig. 23 CV curves of catalysts. (a), COF-205-Ru; (b), RuO_2 and (c), IrO_2.

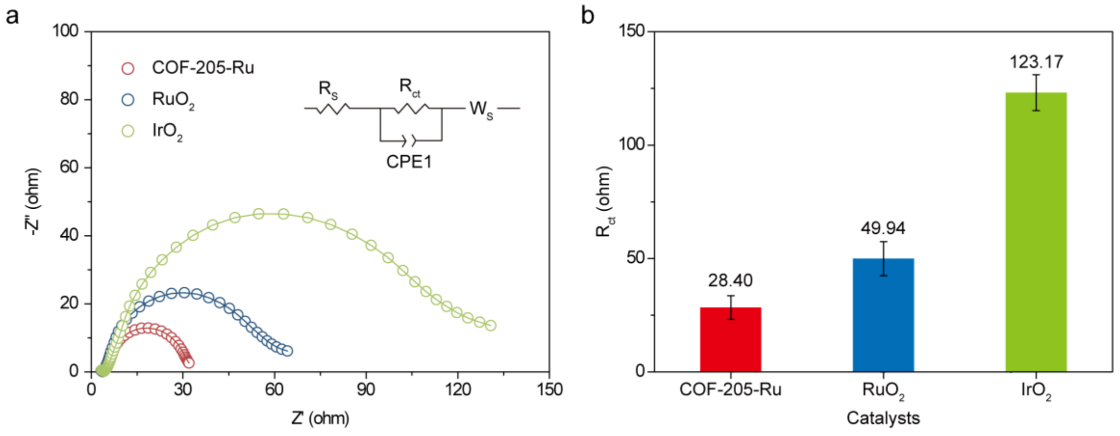

Supplementary Fig. 24 Electrochemical resistance evaluation of catalysts. (a), Electrochemical impedance spectroscopy of the COF-205-Ru, RuO_2, and IrO_2 catalysts (Inset: equivalent fitted circuit diagram); (b), fitted R_{ct} values.

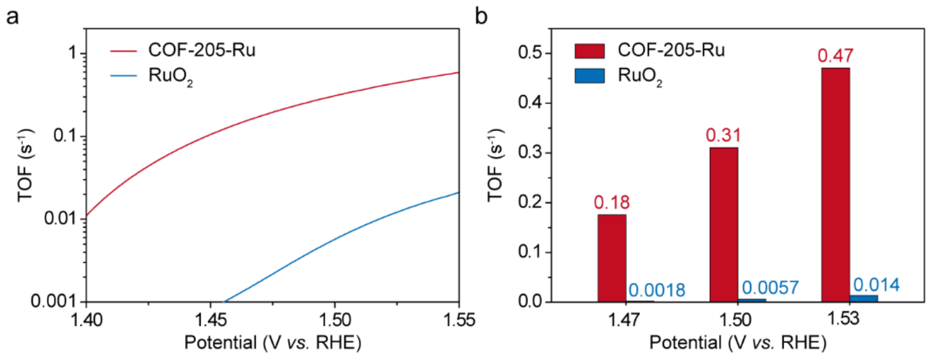

Supplementary Fig. 25 Comparison of TOF values of COF-205-Ru and commercial RuO_2 reference. (a) TOF curves; (b) TOF values at 1.47 V, 1.50 V and 1.53 V (*vs*. RHE), respectively.

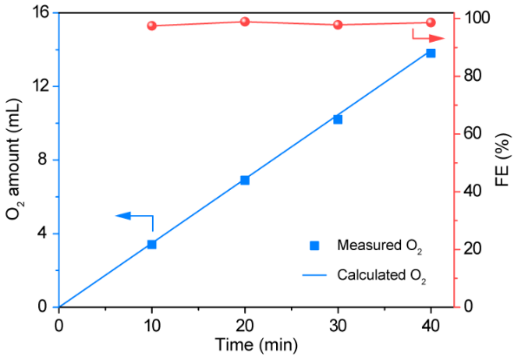

Supplementary Fig. 26 The Faradaic efficiency of COF-205-Ru under 0.5 M H_2SO_4.

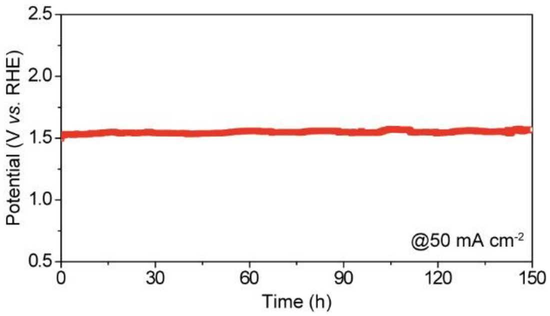

Supplementary Fig. 27 Chronopotentiometry measurements of COF-205-Ru on carbon cloth electrode at current density of 50 mA cm^{-2}.

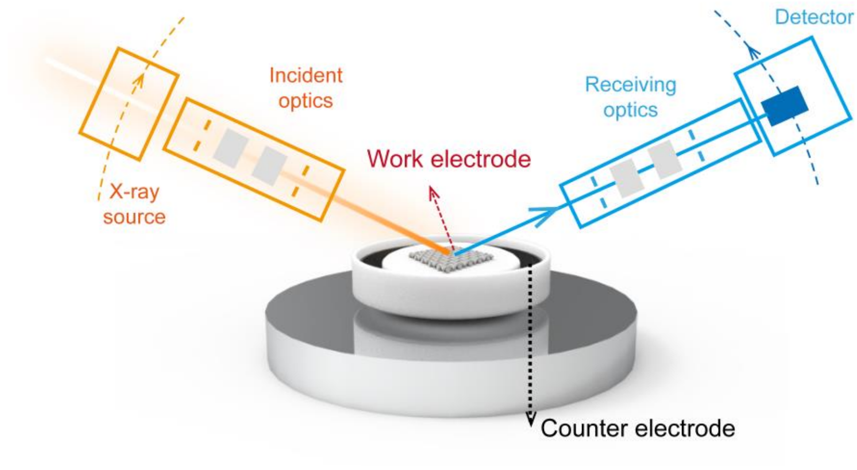

Supplementary Fig. 28 The illustration of *in situ* PXRD measurement.

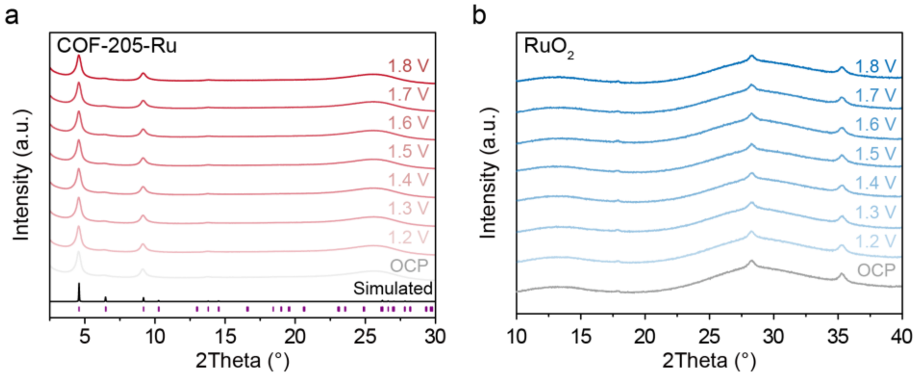

Supplementary Fig. 29 *In situ* PXRD patterns at different applied potentials. (a), COF-205-Ru; (b), commercial RuO_2.

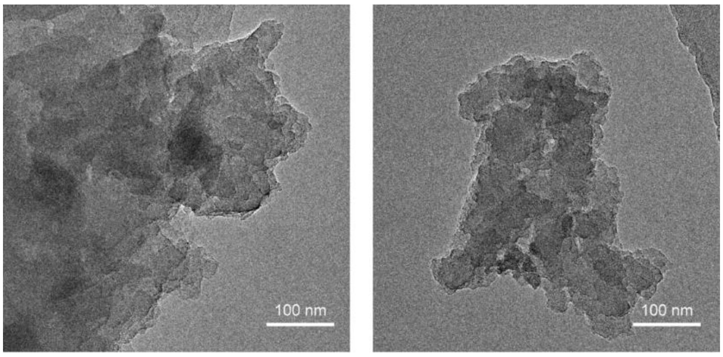

Supplementary Fig. 30 TEM images of COF-205-Ru after OER stability test in 0.5 M H_2SO_4 electrolyte.

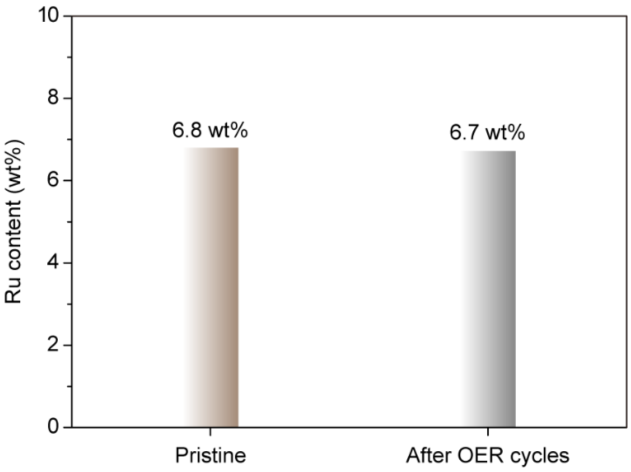

Supplementary Fig. 31 The ICP-OES results of pristine and after stability tested COF-205-Ru.

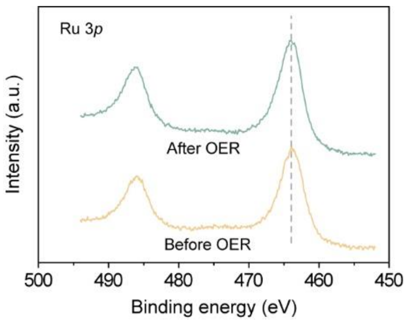

Supplementary Fig. 32 High-resolution XPS of Ru 3*p* for COF-205-Ru before and after OER.

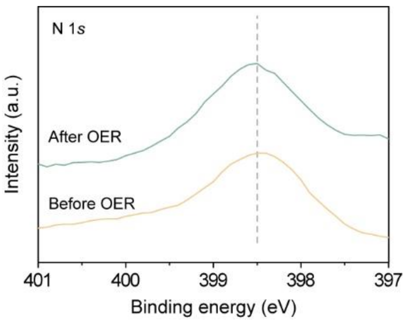

Supplementary Fig. 33 High-resolution XPS of N 1*s* for COF-205-Ru before and after OER.

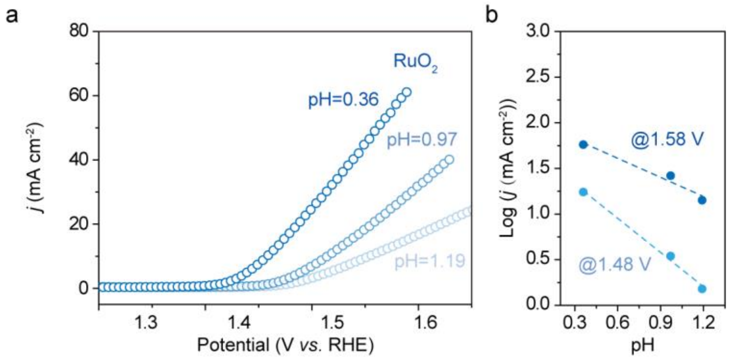

Supplementary Fig. 34 The pH-dependence of catalytic performance on commercial RuO_2. (a) *iR*-corrected LSVs of commercial RuO_2 in H_2SO_4 electrolytes (pH values: 0.36, 0.97 and 1.19, respectively); (b) corresponding log(*j*) at 1.48 V and 1.58 V under different pH values.

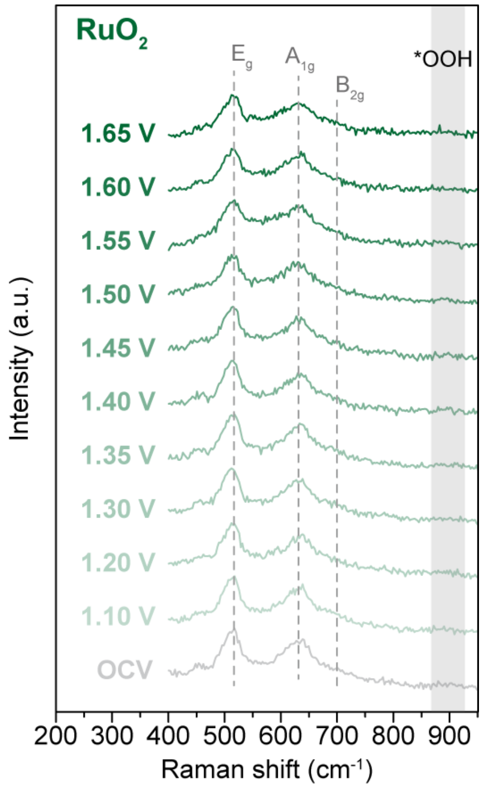

Supplementary Fig. 35 *In situ* Raman spectra of RuO_2 under 0.5 M H_2SO_4 electrolyte at different applied potentials.

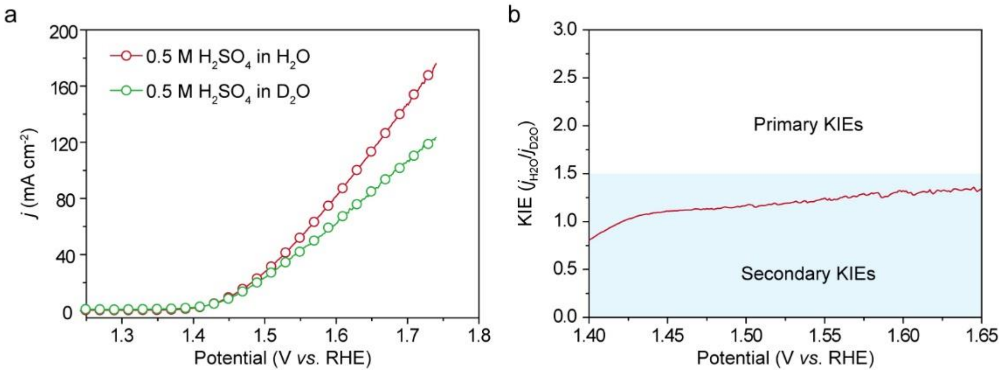

Supplementary Fig. 36 The kinetic isotope effects investigation by H/D isotope-labelled experiment. (a) Polarization curves of COF-205-Ru in 0.5 M H_2SO_4 dissolved in H_2O or D_2O without *iR*-compensation. (b) The corresponding KIE values at different overpotentials.

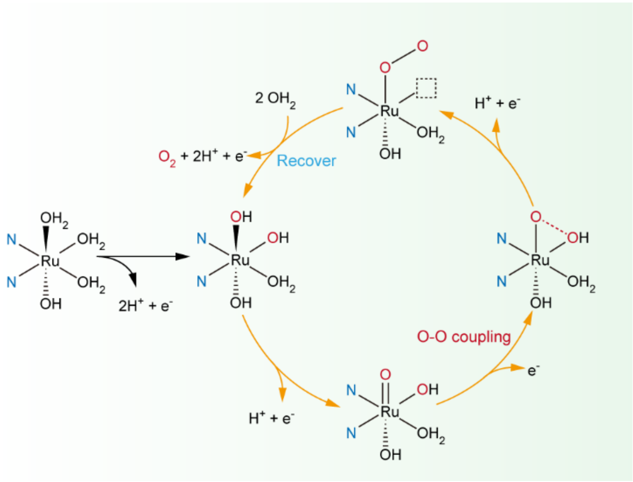

Supplementary Fig. 37 Schematic illustration of the reaction pathway for COF-205-Ru catalyst.

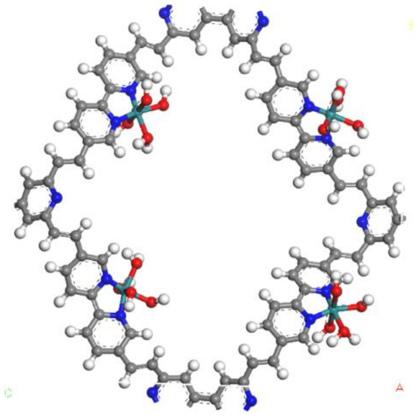

Supplementary Fig. 38 The geometric configuration on the calculated COF-205-Ru model.

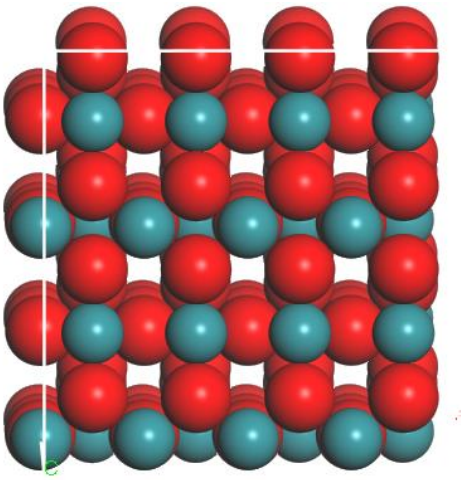

Supplementary Fig. 39 The geometric configuration on the calculated RuO_2 model.

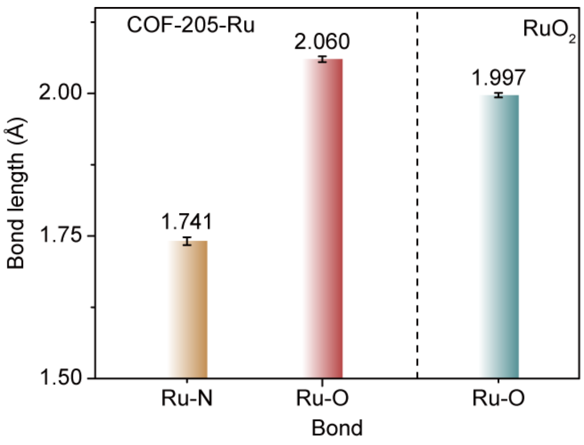

Supplementary Fig. 40 Comparison of the typically calculated bond length for COF-205-Ru and RuO_2.

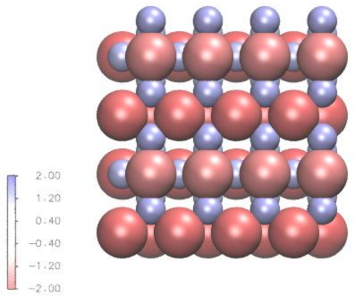

Supplementary Fig. 41 The calculated Bard charge analysis for RuO_2 model.

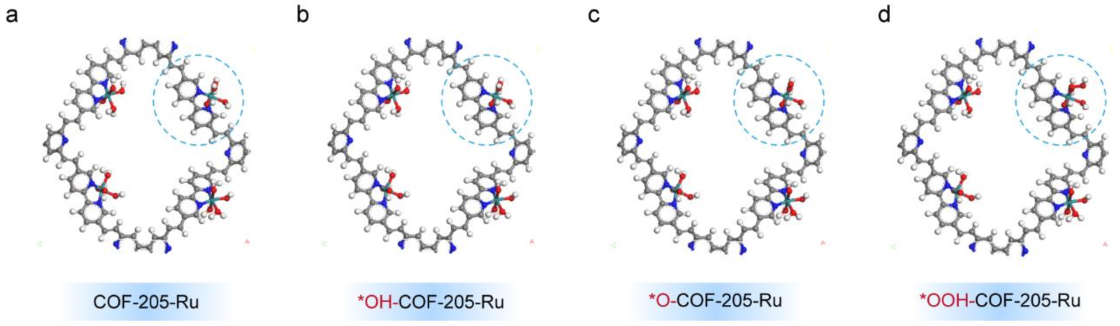

Supplementary Fig. 42 The geometric configuration of COF-205-Ru following AEM pathway. Different adsorbed species: (a), $*OH_2$; (b) OH*; (c) *O; and (d) *OOH on Ru site. The dark cyan, light grey, blue, red, and white balls represent Ru, C, N, O, and H atoms, respectively.

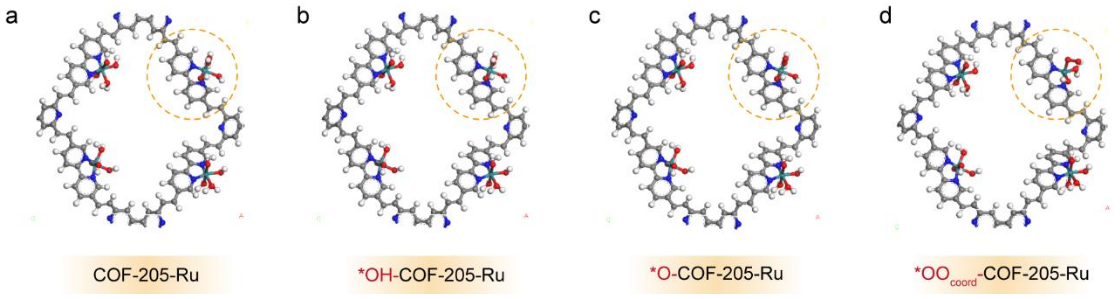

Supplementary Fig. 43 The geometric configuration of COF-205-Ru following IHNA pathway. Different adsorbed species: (a), $*OH_2$; (b) OH*; (c) *O; and (d) $*OO_{coord}$ on Ru site. The dark cyan, light grey, blue, red, and white balls represent Ru, C, N, O, and H atoms, respectively.

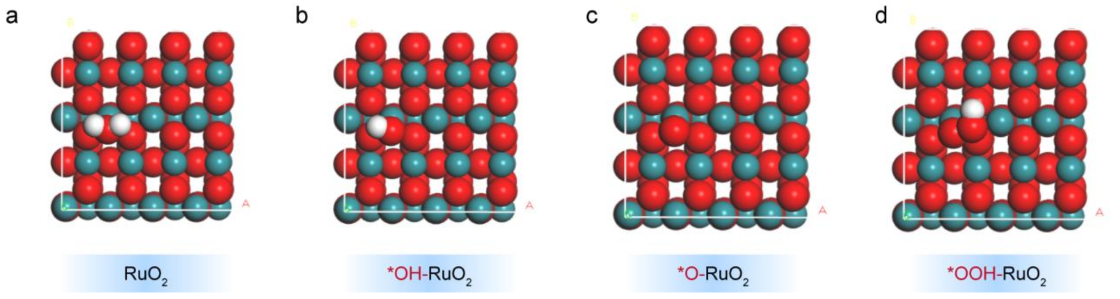

Supplementary Fig. 44 The geometric configuration of RuO_2 following AEM pathway. Different adsorbed species: (a), $*OH_2$; (b) OH*; (c) *O; and (d) *OOH on Ru site. The dark cyan, red, and white balls represent Ru, O, and H atoms, respectively.

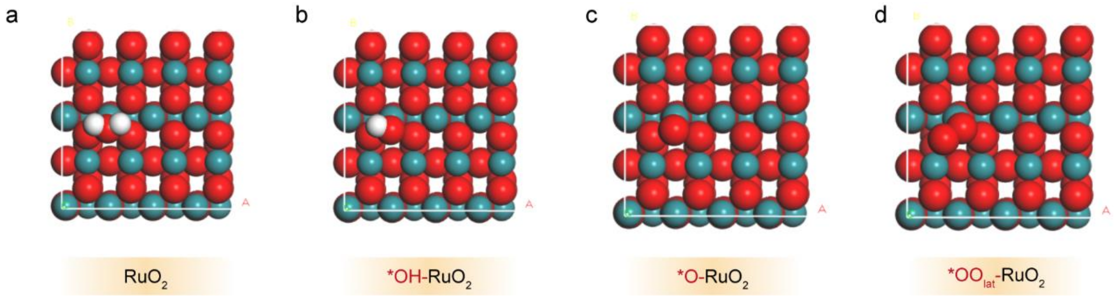

Supplementary Fig. 45 The geometric configuration of RuO_2 following LOM pathway. Different adsorbed species: (a), $*OH_2$; (b) OH*; (c) *O; and (d) $*OO_{lat}$ on Ru site. The dark cyan, red, and white balls represent Ru, O, and H atoms, respectively.

Table S1. Structural parameters extracted from the Ru K-edge EXAFS fitting. (S_0^2=0.70).

Sample	Path	CN	R (Å)	σ^2 (10^{-3} Å^2)	ΔE_0 (eV)	R factor
Ru foil	Ru-Ru	12*	2.67±0.02	2.6	4.4	0.01
RuO_2	Ru–O	5.6±0.8	1.99±0.03	3.4	-0.8	0.02
$RuCl_3$	Ru-Cl	5.7±0.9	2.34±0.02	4.3	-2.96	0.02
COF-205-Ru	Ru–N	1.7±0.3	2.05±0.03	1.0	9.3	0.02
	Ru–O	3.4±0.6	2.19±0.02	1.7	9.3	0.02

For the EXAFS fitting, S_0^2 is the amplitude reduction factor; CN is the coordination number; R is interatomic distance (the bond length between Ru central atoms and surrounding Ru atoms or coordination oxygen atoms); σ^2 is Debye-Waller factor (a measure of thermal and static disorder in absorber-scatter distances); ΔE_0 is edge-energy shift (the difference between the zero kinetic energy value of the sample and that of the theoretical model). R factor is used to value the goodness of the fitting. $S0^2$ was fixed according to the experimental EXAFS fit of Ru foil by fixing CN as the known crystallographic value.

Table 2. Comparison of representative Ru-based OER catalysts in 0.5 M H_2SO_4 electrolyte.

Catalyst	j (mA cm^{-2})	η (mV)	Stability	Reference
**COF-205-Ru**	**10**	**212**	**125 h**	**This work**
**RuO_2**	**10**	**281**	**10 h**	**This work**
1D-RuO_2-CN_x	10	250	50 h	1
RuO_2/Co_3O_4−RuCo@NC	10	247	8 h	2
$Y_{1.85}Zn_{0.15}Ru_2O_{7-\delta}$	10	290	8.5 h	3
$Y_{1.85}Ba_{0.15}Ru_2O_7$	10	278	4 h	4
Mg-doping RuO_2	10	228	30 h	5
$RuNiO_x$	50	280	10 h	6
Fe_3O_4/RuO_2@NEU-7	10	450	-	7
Ru/RuO_2@N-rGO	10	260	-	8
$Y_{1.7}Sr_{0.3}Ru_2O_7$	10	264	28	9
C-RuO_2-RuSe-10	10	242	50 h	10
Ru_1-Pt_3Cu	10	220	28 h	11
Ru@IrO_x	10	282	24 h	12
$RuNi_2$@G250	10	210	3 h	13
PtCo-RuO_2	10	216	24 h	14
UfD- RuO_2	10	179	20 h	15
Vo- RuO_2 NSs	10	225	6 h	16
RuCu NSs	10	236	12 h	17
P-Ru/Fe NAs	10	238	9 h	18
Ru-N-C	10	267	30 h	19
RuIr-NC	10	255	120 h	20
Co-SAC/ RuO_2	10	200	20 h	21
SS Pt- RuO_2	10	228	100 h	22

Table 3. Comparison of mass activity for representative OER catalysts.

Catalyst	Mass activity (A g_{metal}^{-1})	Reference
**COF-205-Ru**	**2659.3@320 mV**	**This work**
**RuO_2**	**82.3@320 mV**	**This work**
**IrO_2**	**23.5@320 mV**	**This work**
Ir-CCTO	1195@300 mV	23
py-RuO_2:Zn	881@300 mV	24
RuO_2-WC NPs	1430@320 mV	25
Li_xRuO_2	~253@300 mV	26
S_H-RuCuO NRs	783@270 mV	27
Ru/α-MnO_2	~431@161 mV	28
Na-a/c-RuO_2	~100@250 mV	29
Ni-Ru@RuO_x	315@220 mV	30
$In_{0.17}Ru_{0.83}O_2$	1094.9@300 mV	31
defect-rich RuO_2 nanosheets	520@230 mV	16
$W_{0.2}Er_{0.1}Ru_{0.7}O_{2-\delta}$	1518.6@275 mV	32
$RuNi_2$@G-250	57.6@250 mV	13
Mn-RuO_2	596.38@270 mV	33
$SrIr_2O_6$	61.3@300 mV	34
Ir NSs	221.8@300 mV	35
Cu-doped RuO_2	352@350 mV	36
Ru@IrOx	644.8@330 mV	12

References

1 Bhowmik, T., Kundu, M. K. & Barman, S. Growth of one-dimensional RuO_2 nanowires on g-carbon nitride: An active and stable bifunctional electrocatalyst for hydrogen and oxygen evolution reactions at all pH values. *ACS Appl. Mater. Interfaces* **8**, 28678-28688 (2016).

2 Fan, Z., Jiang, J., Ai, L., Shao, Z. & Liu, S. Rational design of ruthenium and cobalt-based composites with rich metal-insulator interfaces for efficient and stable overall water splitting in acidic electrolyte. *ACS Appl. Mater. Interfaces* **11**, 47894-47903 (2019).

3 Feng, Q. et al. Highly active and stable ruthenate pyrochlore for enhanced oxygen evolution reaction in acidic medium electrolysis. *Appl. Catal., B* **244**, 494-501 (2019).

4 Feng, Q. et al. Influence of surface oxygen vacancies and ruthenium valence state on the catalysis of pyrochlore oxides. *ACS Appl. Mater. Interfaces* **12**, 4520-4530 (2020).

5 Li, Y. et al. Hollow IrCo nanoparticles for high-performance overall water splitting in an acidic medium. *ACS Appl. Nano Mater.* **3**, 11916-11922 (2020).

6 An, L. et al. Dealloyed $RuNiO_x$ as a robust electrocatalyst for the oxygen evolution reaction in acidic media. *Dalton Trans.* **50**, 5124-5127 (2021).

7 Fonseca, J. & Choi, S. Electro- and photoelectro-catalysts derived from bimetallic amorphous metal–organic frameworks. *Catal. Sci. Technol.* **10**, 8265-8282 (2020).

8 Gao, X. et al. Ru/RuO_2 nanoparticle composites with N-doped reduced graphene oxide as electrocatalysts for hydrogen and oxygen evolution. *ACS Appl. Nano Mater.* **3**, 12269-12277 (2020).

9 Zhang, N. et al. Metal substitution steering electron correlations in pyrochlore ruthenates for efficient acidic water oxidation. *ACS Nano* **15**, 8537-8548 (2021).

10 Wang, J. et al. Exceptionally active and stable RuO_2 with interstitial carbon for water oxidation in acid. *Chem* **8**, 1673-1687 (2022).

11 Yeo, B. S. Oxygen evolution by stabilized single Ru atoms. *Nat. Catal.* **2**, 284-285 (2019).

12 Shan, J. et al. Charge-Redistribution-Enhanced Nanocrystalline Ru@IrOx Electrocatalysts for Oxygen Evolution in Acidic Media. *Chem* **5**, 445-459 (2019).

13 Cui, X. et al. Robust interface Ru centers for high-performance acidic oxygen evolution. *Adv. Mater.* **32**, e1908126 (2020).

14 Jin, H. et al. Safeguarding the RuO_2 phase against lattice oxygen oxidation during acidic water electrooxidation. *Energy Environ. Sci.* **15**, 1119-1130 (2022).

15 Ge, R. et al. Ultrafine defective RuO_2 electrocatayst integrated on carbon cloth for robust water oxidation in acidic media. *Adv. Energy Mater.* **9**, 1901313 (2019).

16 Zhao, Z. L. et al. Boosting the oxygen evolution reaction using defect-rich ultra-thin ruthenium oxide nanosheets in acidic media. *Energy Environ. Sci.* **13**, 5143-5151 (2020).

17 Yao, Q. et al. Channel-rich RuCu nanosheets for pH-universal overall water splitting electrocatalysis. *Angew. Chem., Int. Ed.* **58**, 13983-13988 (2019).

18 Yao, Q. et al. A chemical etching strategy to improve and stabilize RuO_2-based nanoassemblies for acidic oxygen evolution. *Nano Energy* **84**, 105909 (2021).

19 Cao, L. et al. Dynamic oxygen adsorption on single-atomic Ruthenium catalyst with high performance for acidic oxygen evolution reaction. *Nat. Commun.* **10**, 4849 (2019).

20 Wu, D. et al. Efficient overall water splitting in acid with anisotropic metal nanosheets. *Nat. Commun.* **12**, 1145 (2021).

21 Shah, K. et al. Cobalt single atom incorporated in ruthenium oxide sphere: A robust bifunctional electrocatalyst for HER and OER. *Angew. Chem., Int. Ed.* **61**, e202114951 (2022).

22 Wang, J. et al. Single-site Pt-doped RuO_2 hollow nanospheres with interstitial C for high-performance acidic overall water splitting. *Sci. Adv.* **8**, eabl9271 (2022).

23 Thao, N. T. T. et al. Colossal dielectric perovskites of calcium copper titanate ($CaCu_3Ti_4O_{12}$) with low-iridium dopants enables ultrahigh mass activity for the acidic oxygen evolution reaction. *Adv. Sci.* **10**, e2207695 (2023).

24 Zhang, D. et al. Construction of Zn-doped RuO_2 nanowires for efficient and stable water oxidation in acidic media. *Nat. Commun.* **14**, 2517 (2023).

25 Sun, S. C. et al. Bifunctional WC-supported RuO_2 nanoparticles for robust water splitting in acidic media. *Angew. Chem., Int. Ed.* **61**, e202202519 (2022).

26 Qin, Y. et al. RuO_2 electronic structure and lattice strain dual engineering for enhanced acidic oxygen evolution reaction performance. *Nat. Commun.* **13**, 3784 (2022).

27 Yao, Q. et al. S incorporated RuO_2-based nanorings for active and stable water oxidation in acid. *Nano Res.* **15**, 3964-3970 (2022).

28 Lin, C. et al. *In-situ* reconstructed Ru atom array on α-MnO_2 with enhanced performance for acidic water oxidation. *Nat. Catal.* **4**, 1012-1023 (2021).

29 Zhang, L. et al. Sodium-decorated amorphous/crystalline RuO_2 with rich oxygen vacancies: A robust pH-universal oxygen evolution electrocatalyst. *Angew. Chem., Int. Ed.* **60**, 18821-18829 (2021).

30 Harzandi, A. M. et al. Ruthenium core–shell engineering with nickel single atoms for selective oxygen evolution via nondestructive mechanism. *Adv. Energy Mater.* **11**, 2003448 (2021).

31 Chen, S. et al. An indium-induced-synthesis $In_{0.17}Ru_{0.83}O_2$ nanoribbon as highly active electrocatalyst for oxygen evolution in acidic media at high current densities above 400 mA cm^{-2}. *J. Mater. Chem. A* **10**, 3722-3731 (2022).

32 Hao, S. et al. Dopants fixation of Ruthenium for boosting acidic oxygen evolution stability and activity. *Nat. Commun.* **11**, 5368 (2020).

33 Chen, S. et al. Mn-doped RuO_2 nanocrystals as highly active electrocatalysts for enhanced oxygen evolution in acidic media. *ACS Catal.* **10**, 1152-1160 (2019).

34 Wang, L. et al. Structurally robust honeycomb layered strontium iridate as an oxygen evolution electrocatalyst in acid. *ACS Catal.* **13**, 7322-7330 (2023).

35 Wu, G. et al. A general synthesis approach for amorphous noble metal nanosheets. *Nat. Commun.* **10**, 4855 (2019).

36 Su, J. et al. Assembling ultrasmall copper-doped ruthenium oxide nanocrystals into hollow porous polyhedra: highly robust electrocatalysts for oxygen evolution in acidic media. *Adv. Mater.* **30**, e1801351 (2018).
