# Supplementary material for: Stabilizing atomic Ru species in conjugated sp2 carbon-linked covalent organic framework for acidic water oxidation
Source: Nat Commun. 2024 Jun 26;15:5419. doi: 10.1038/s41467-024-49834-5 (PMC11208516; doi:10.1038/s41467-024-49834-5)
Supplement: Supplementary file 3 — Description of Additional Supplementary Files [file 41467_2024_49834_MOESM3_ESM.pdf]

Description of Supplementary Data Files

Supplementary Data 1: The atomic coordinates of the optimized computational models for RuO_2.

Supplementary Data 2: The atomic coordinates of the optimized computational models for COF-205-Ru.
